# Gradient estimation in dendritic reinforcement learning

**DOI:** 10.1186/2190-8567-2-2

**Published:** 2012-02-15

**Authors:** Mathieu Schiess, Robert Urbanczik, Walter Senn

**Affiliations:** 1 Department of Physiology, University of Bern, Bühlplatz 5, 3012, Bern, Switzerland

**Keywords:** Dendritic computation, reinforcement learning, spiking neuron

## Abstract

We study synaptic plasticity in a complex neuronal cell model where NMDA-spikes can arise in certain dendritic zones. In the context of reinforcement learning, two kinds of plasticity rules are derived, zone reinforcement (ZR) and cell reinforcement (CR), which both optimize the expected reward by stochastic gradient ascent. For ZR, the synaptic plasticity response to the external reward signal is modulated exclusively by quantities which are local to the NMDA-spike initiation zone in which the synapse is situated. CR, in addition, uses nonlocal feedback from the soma of the cell, provided by mechanisms such as the backpropagating action potential. Simulation results show that, compared to ZR, the use of nonlocal feedback in CR can drastically enhance learning performance. We suggest that the availability of nonlocal feedback for learning is a key advantage of complex neurons over networks of simple point neurons, which have previously been found to be largely equivalent with regard to computational capability.

## 1 Introduction

 Except for biologically detailed modeling studies, the overwhelming majority of works in mathematical neuroscience have treated neurons as point neurons, i.e., a linear aggregation of synaptic input followed by a nonlinearity in the generation of somatic action potentials was assumed to characterize a neuron. This disregards the fact that many neurons in the brain have complex dendritic arborization where synaptic inputs may be aggregated in highly nonlinear ways [1]. From an information processing perspective sticking with the minimal point neuron may nevertheless seem justified since networks of such simple neurons already display remarkable computational properties: assuming infinite precision and noiseless arithmetic a suitable network of spiking point neurons can simulate a universal Turing machine and, further, impressive information processing capabilities persist when one makes more realistic assumptions such as taking noise into account (see [2] and the references therein). Such generic observations are underscored by the detailed compartmental modeling of the computation performed in a hippocampal pyramidal cell [3]. There it was found that (in a rate coding framework) the input-output behavior of the complex cell is easily emulated by a simple two layer network of point neurons.

 If the computations of complex cells are readily emulated by relatively simple circuits of point neurons, the question arises why so many of the neurons in the brain are complex. Of course, the reason for this may be only loosely related to information processing proper, it might be that maintaining a complex cell is metabolically less costly than the maintenance of the equivalent network of point neurons. Here, we wish to explore a different hypothesis, namely that complex cells have crucial advantages with regard to learning. This hypothesis is motivated by the fact that many artificial intelligence algorithms for neural networks assume that synaptic plasticity is modulated by information which arises far downstream of the synapse. A prominent example is the backpropagation algorithm where error information needs to be transported upstream via the transpose of the connectivity matrix. But in real axons any fast information flow is strictly downstream, and this is why algorithms such as backpropagation are widely regarded as a biologically unrealistic for networks of point neurons. When one considers complex cells, however, it seems far more plausible that synaptic plasticity could be modulated by events which arise relatively far downstream of the synapse. The backpropagating action potential, for instance, is often capable of conveying information on somatic spiking to synapses which are quite distal in the dendritic tree [4,5]. If nonlinear processing occurred in the dendritic tree during the forward propagation, this means that somatic spiking can modulate synaptic plasticity even when one or more layers of nonlinearities lie between the synapse and the soma. Thus, compared to networks of point neurons, more sophisticated plasticity rules could be biologically feasible in complex cells.

**Fig. 1:**
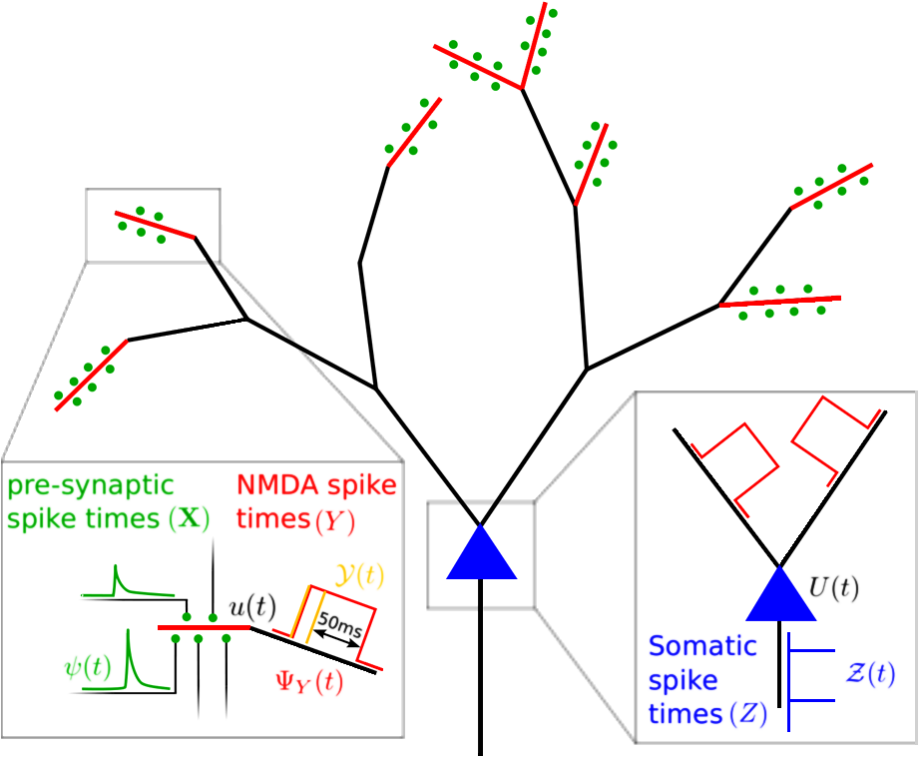
Sketch of the neuronal cell model. Spatio-temporally clustered postsynaptic potentials (PSP, *green*) can give rise to NMDA-spikes (*red*) which superimpose additively in the soma (*blue*) controlling the generation of action potentials (AP).

 To study this issue, we formalize a complex cell as a two layer network, with the first layer made up of initiation zones for NMDA-spikes (Figure 1). NMDA-spikes are regenerative events, caused by AMPA mediated synaptic releases when the releases are both near coincident in time and spatially co-located on the dendrite [6-8]. Such NMDA-spikes boost the effect of the synaptic releases, leading to increases in the somatic potential which are stronger as well as longer compared to the effect obtained from a simple linear superposition of the excitatory post synaptic potentials from the individual AMPA releases. Further, we assume that the contribution of NMDA-spikes from different initiation zones combine additively in contributing to the somatic potential and that this potential governs the generation of somatic action potentials via an escape noise process. While we would argue that this provides an adequate minimal model of dendritic computation in basal dendritic structures, one should bear in mind that our model seems insufficient to describe the complex interactions of basal and apical dendritic inputs in cortical pyramidal cells [9,10]. 

 We will consider synaptic plasticity in the context of reinforcement learning, where the somatic action potentials control the delivery of an external reward signal. The goal of learning is to adjust the strength of the synaptic releases (the synaptic weights) so as to maximize the expected value of the reward signal. In this framework, one can mathematically derive plasticity rules [11,12] by assuming that weight adaption follows a stochastic gradient ascent procedure in the expected reward [13]. Dopamine is widely believed to be the most important neurotransmitter for such reward modulated plasticity [14-16]. A simple minded application of the approach in [13] leads to a learning rule where, except for the external reward signal, plasticity is determined by quantities which are local to each NMDA-spike initiation zone (NMDA-zone). Using this rule, NMDA-zones learn as independent agents which are oblivious of their interaction in generating somatic action potentials, with the external reward signal being the only mechanism for coordinating plasticity between the zones. hence we shall refer to this rule as zone reinforcement (ZR). Due to its simplicity, ZR would seem biologically feasible even if the network were not integrated into a single neuron. On the other hand, this approach to multi-agent reinforcement often leads to a learning performance which deteriorates quickly as the number of agents (here, NMDA-zones) increases since it lacks an explicit mechanism for differentially assigning credit to the agents [17,18]. By algebraic manipulation of the gradient formula leading to the basic ZR-rule, we derive a class of learning rules where synaptic plasticity is also modulated by somatic responses, in addition to reward and quantities local to the NMDA-zone. Such learning rules will be referred to as cell reinforcement (CR), since they would be biologically unrealistic if the nonlinearities where not integrated into a single cell. We present simulation result showing that one rule in the CR-class results in learning which is much faster than for the ZR-rule. This provides evidence for the hypothesis that enabling effective synaptic plasticity rules may be one evolutionary advantage conveyed by dendritic nonlinearities.

## 2 Stochastic cell model of a neuron

We assume a neuron with N=40 initiation zones for NMDA-spikes, indexed by ν=1,…,N. An NMDA-zone is made up of $M_{\nu }$ synapses, with synaptic strength $w_{i,\nu }$ ($i=1,\ldots ,M_{\nu }$), where releases are triggered by presynaptic spikes. We denote by $X_{i,\nu }$ the set of times when presynaptic spikes arrive at synapse (i,ν). In each NMDA-zone, the synaptic releases give rise to a time varying local membrane potential $u_{\nu }$ which we assume to be given by a standard spike response equation

(1) $$u_{\nu }(t;X)=U_{\mathrm{rest}}+\sum_{i}^{M_{\nu }}w_{i,\nu }\sum_{s\in X_{i,\nu }}\epsilon (t-s).$$

Here, **X** denotes the entire presynaptic input pattern of the neuron, $U_{\mathrm{rest}}=-1$ (arbitrary units) is the resting potential, and the postsynaptic response kernel *ϵ* is given by

$$\varepsilon (t)=\frac{\Theta (t)}{\tau_{m}-\tau_{s}}(e^{-t/\tau_{m}}-e^{-t/\tau_{s}}).$$

We use $\tau_{m}=10\text{ ms}$ for the membrane time constant, $\tau_{s}=1.5\text{ ms}$ for the synaptic rise time, and Θ is the Heaviside step function.

 The local potential $u_{\nu }$ controls the rate at which what we call NMDA-events are generated in the zone - in our model NMDA-events are closely related to the onset of NMDA-spikes as described in detail below. Formally, we assume that NMDA-events are generated by an inhomogeneous Poisson process with rate function $\phi_{N}(u_{\nu }(t;X))$, choosing

(2) $$\phi_{N}(x)=q_{N}e^{\beta_{N}x}$$

with $q_{N}=0.005$ and $\beta_{N}=3$. We adopt the symbol $Y^{\nu }$ to denote the set of NMDA-event times in zone *ν*. For future use, we recall the standard result [19] that the probability density $P_{w_{\cdot ,\nu }}(Y^{\nu }|X)$ of an event-train $Y^{\nu }$ generated during an observation period running from t=0 to *T* satisfies

(3) $$\log P_{w_{\cdot ,\nu }}(Y^{\nu }|X)=\int_{0}^{T}dt\;\log\left(q_{N}e^{\beta_{N}u_{\nu }(t;X)}\right)Y^{\nu }(t)-q_{N}e^{\beta_{N}u_{\nu }(t;X)},$$

where $Y^{\nu }(t)=\sum_{s\in Y^{\nu }}\delta (t-s)$ is the *δ*-function representation of $Y^{\nu }$.

Conceptually, it would be simplest to assume that each NMDA-event initiates a NMDA-spike. But we need some mechanism for refractoriness, since NMDA-spikes have an extended duration (20-200 ms) and there is no evidence that multiple simultaneous NMDA-spikes can arise in a single NMDA-zone. Hence, we shall assume that, while a NMDA-event occurring in temporal isolation causes a NMDA-spike, a rapid succession of NMDA-events within one zone only leads to a somewhat longer but not to a stronger NMDA-spike. In particular, we will assume that a NMDA-spike contributes to the somatic potential during a period of Δ=50 ms after the time of the last preceding NMDA-event. Hence, if a NMDA-event is followed by a second one with a 5 ms delay, the first event initiates a NMDA-spike which lasts for 55 ms due to the second NMDA-event. Formally, we denote by $s_{Y^{\nu }}(t)=\max\{s\leq t|s\in Y^{\nu }\}$ the time of the last NMDA-event up to time *t* and model the somatic effect of an NMDA-spike by the response kernel

(4) $$\Psi_{Y^{\nu }}(t)=\{\begin{array}{cc} 1 & \text{if }0\leq t-s_{Y^{\nu }}(t)\leq \Delta =50\text{ ms},\\ 0 & \text{otherwise.} \end{array}$$

The main motivation for modeling the generation of NMDA-spikes in this way is that it proves mathematically convenient in the calculations below. Having said this, it is worthwhile mentioning that treating NMDA-spikes as rectangular pulses seems reasonable, since their rise and fall times are typically short compared to the duration of the spike. Also, there is some evidence that increased excitatory presynaptic activity extends the duration of a NMDA-spike but does not increase its amplitude [7,8]. Qualitatively, the above model is in line with such findings.

For specifying the somatic potential *U* of the neuron, we denote by **Y** the vector of all NMDA-event trains $Y^{\nu }$ and by *Z* the set of times when the soma generates action potentials. We then use

(5) $$U(t;Y,Z)=U_{\mathrm{rest}}+\sum_{\nu =1}^{N}a\Psi_{Y^{\nu }}(t)-\sum_{s\in Z}\kappa (t-s)$$

for the time course of the somatic potential, where the reset kernel *κ* is given by

$$\kappa (t)=\Theta (t)e^{-t/\tau_{m}}.$$

This is a highly stylized model of the somatic potential since we assume that NMDA-zones contribute equally to the somatic potential (with a strength controlled by the positive parameter *a*) and that, further, the AMPA-releases themselves do not contribute directly to *U*. Even if these restrictive assumptions may not be entirely unreasonable (for instance, AMPA-releases can be much more strongly attenuated on their way to the soma than NMDA-spikes) we wish to point out that, while becoming simpler, the mathematical approach below does not rely on these restrictions.

 Somatic firing is modeled as an escape noise process with an instantaneous rate function $\phi_{S}(U(t;Y,Z))$ where

(6) $$\phi_{S}(x)=q_{S}e^{\beta_{S}x}$$

with $q_{S}=0.005$ and $\beta_{S}=5$. As shown in [20], for the probability density P(Z|Y) of responding to the NMDA-events with a somatic spike train *Z* during the observation period this implies

(7) $$\log P(Z|Y)=\int_{0}^{T}dt\;\log\left(q_{S}e^{\beta_{S}U(t;Z,Y)}\right)Z(t)-q_{S}e^{\beta_{S}U(t;Z,Y)}$$

with $Z(t)=\sum_{s\in Z}\delta (t-s)$.

## 3 Reinforcement learning

In reinforcement learning, one assumes a scalar reward function R(Z,X) providing feedback about the appropriateness of the somatic response *Z* to the input **X**. The goal of learning is to adapt the synaptic strengths so as to obtain appropriate somatic responses. For our neuronal model, the expected value $\bar{R}$ of the reward signal R(Z,X) is

(8) $$\bar{R}(w)=\int dX\;dY\;dZ\;P(X)P_{w}(Y|X)P(Z|Y)R(Z,X),$$

where P(X) is the probability density of the input spike patterns and $P_{w}(Y|X)=\prod_{\nu =1}^{N}P_{w_{\cdot ,\nu }}(Y^{\nu }|X)$. The goal of learning can now be formalized as finding a **w** maximizing $\bar{R}$ and synaptic plasticity rules can be obtained using stochastic gradient ascent procedures for this task.

 In stochastic gradient ascent, X,Y, and *Z* are sampled at each trial and every weight is updated by

$$w_{i,\nu }\leftarrow w_{i,\nu }+\eta g_{i,\nu }(X,Y,Z),$$

where η>0 is the learning rate and $g_{i,\nu }(X,Y,Z)$ is an (unbiased) estimator of $\frac{\partial }{\partial w_{i,\nu }}\bar{R}$. Under mild regularity conditions, convergence to a local optimum is guaranteed if one uses an appropriate schedule for decreasing *η* towards 0 during learning [21]. In biological modeling, one usually simply assumes a small but fixed learning rate.

The derivative of $\bar{R}$ with respect to the weight of synapse (i,ν) can be written as

(9) $$\frac{\partial }{\partial w_{i,\nu }}\bar{R}=\int dX\;dY\;dZ\;P(X)P_{w}(Y|X)P(Z|Y)R(Z,X)\frac{\partial }{\partial w_{i,\nu }}\log P_{w_{\cdot ,\nu }}(Y^{\nu }|X).$$

Hence, a simple choice for the gradient estimator is

(10) $$g_{i,\nu }^{\mathrm{ZR}}(X,Y,Z)=R(Z,X)\frac{\partial }{\partial w_{i,\nu }}\log P_{w_{\cdot ,\nu }}(Y^{\nu }|X)$$

with $P_{w_{\cdot ,\nu }}(Y^{\nu }|X)$ given by Equation 3. Note that the conditional probability P(Z|Y) does not explicitly appear in the estimator, so the update is oblivious of the architecture of the model neuron, i.e., of how NMDA-events contribute to somatic spiking. Since the only learning mechanism for coordinating the responses of the different NMDA-zones is the global reward signal R(Z,X), we refer to the update given by Equation 10 as ZR.

Better plasticity rules can be obtained by algebraic manipulations of Equations 8 and 9 which yield gradient estimators which have a reduced variance compared to Equation 10 - this should lead to faster learning. A simple and well-known example for this is adjusting the reinforcement baseline by choosing a constant *c* and replacing R(Z,X) with R(Z,X)+c in Equation 10; this amounts to adding *c* to $\bar{R}(w)$ and hence does not change the gradient. But a judicious choice of *c* can reduce the variance of the gradient estimator. More ambitiously, one could consider analytically integrating out **Y** in Equation 8, yielding an estimator which directly considers the relationship between synaptic weights and somatic spiking because it is based on $\frac{\partial }{\partial w_{i,\nu }}\log P_{w}(Z|X)$. While actually doing the integration analytically seems impractical, we shall obtain estimators below from a partial realization of this program.

## 4 From zone reinforcement to cell reinforcement

Due to the algebraic symmetries of our model cell, it suffices to give explicit plasticity rules only for one synaptic weight. To reduce clutter we will thus focus on the first synapse $w_{1,1}$ in the first NMDA-zone.

### 4.1 Notational simplifications

Let $Y^{\setminus }$ denote the vector $\left(Y^{2},\ldots ,Y^{N}\right)$ of all NMDA-event trains but the first and $w^{\setminus }$ the collection of synaptic weights $\left(w_{.,2},\ldots ,w_{.,N}\right)$ in all but the first NMDA-zone. We rewrite the expected reward as

(11) $$\begin{array}{rcl} \bar{R}(w) & = & \int dX\;dY^{\setminus }\;P(X)P_{w^{\setminus }}(Y^{\setminus }|X)r(w_{\cdot ,1},X,Y^{\setminus })\;\text{with}\\ r(w_{\cdot ,1},X,Y^{\setminus }) & = & \int dZ\;dY^{1}\;P(Z|Y)P_{w_{\cdot ,1}}(Y^{1}|X)R(Z,X). \end{array}$$

Since in Equation 11 only *r* depends on $w_{1,1}$ we just need to consider $\frac{\partial }{\partial w_{1,1}}r$. Hence, we can regard **X** and $Y^{\setminus }$ as fixed and suppress them in the notation. This allows us to write the somatic potential (Equation 5) simply as

(12) $$U(t;Z,Y)=U_{\mathrm{base}}(t;Z)+a\Psi_{Y}(t)$$

using *Y* as shorthand for the NMDA-event train $Y^{1}$ of the first zone and, further, incorporating into a time varying base potential $U_{\mathrm{base}}$ the following contributions in Equation 5: (i) the resting potential, (ii) the influence of $Y^{\setminus }$, i.e., NMDA-events in the other zones, (iii) any reset caused by somatic spiking. Similarly, the notation for the local membrane potential of the first NMDA-zone becomes

(13) $$u(t)=u_{\mathrm{base}}(t)+w\psi (t),$$

where *w* stands for the strength $w_{1,1}$ of the first synapse, $\psi (t)=\sum_{s\in X_{1,1}}\epsilon (t-s)$, and the effect of the other synapses impinging on the zone is absorbed into $u_{\mathrm{base}}(t)$. Finally, the *w*-dependent contribution *r* to the expected reward (Equation 11) can be written as

(14) $$r(w)=\int dZ\;dY\;P(Z|Y)P_{w}(Y)R(Z),$$

where also for *R* and $P_{w}$ we have suppressed the dependence on *X*. In the reduced notation, the explicit expression (obtained from Equations 3 and 10) for the gradient estimator in ZR-learning is

(15) $$g^{\mathrm{ZR}}(Y,Z)=R(Z)\int_{0}^{T}dt\;\left(Y(t)-q_{N}e^{\beta_{N}u(t)}\right)\beta_{N}\psi (t).$$

### 4.2 Cell reinforcement

To simplify the manipulation of Equation 14, we replace the Poisson process generating *Y* by a discrete time process with step-size δ>0. We assume that NMDA-events in *Y* can only occur at times $t_{k}=k\delta$ where *k* runs from 1 to K=⌊T/δ⌋ and introduce *K* independent binary random variables $y_{k}\in \{0,1\}$ to record whether or not a NMDA-event occurred. For the probability of not having a NMDA-event at time $t_{k}$ we use

(16) $$P_{w}(y_{k}=0)=e^{-\delta \phi_{N}(u(t_{k}))}.$$

With this definition, we can recover the original Poisson process by taking the limit δ→+0. We use $y=(y_{1},\ldots ,y_{K})$ to denote the entire response of the NMDA-zone and, to make contact with the set-based description of the NMDA-trains, we denote by $\hat{y}$ the set of NMDA-event times in **y**, i.e., $\hat{y}=\{t_{k}|y_{k}=1\}$. Next, the discrete time version of Equation 14 is

(17) $$r_{\delta }(w)=\int dZ\;\sum_{y}R(Z)P(Z|\hat{y})P_{w}(y),$$

where $P_{w}(y)=\prod_{k=1}^{K}P_{w}(y_{k})$. In the end, we will recover *r* from $r_{\delta }$ by taking *δ* to zero.

The derivative of Equation 17 is

$$\frac{\partial }{\partial w}r_{\delta }=\int dZ\;\sum_{y}P(Z|\hat{y})P_{w}(y)R(Z)\sum_{k=1}^{K}\frac{\partial }{\partial w}\log P_{w}(y_{k})$$

and to focus on the contributions to $\frac{\partial }{\partial w}r_{\delta }$ from each time bin we set

(18) $${\mathrm{grad}}_{k}=\int dZ\;\sum_{y}P_{w}(y)P(Z|\hat{y})R(Z)\frac{\partial }{\partial w}\log P_{w}(y_{k}).$$

Hence, $\frac{\partial }{\partial w}r_{\delta }=\sum_{k=1}^{K}{\mathrm{grad}}_{k}$.

We now exploit the trivial fact that we can think of $P(Z|\hat{y})$ as a function linear in $y_{k}$, simply because $y_{k}$ is binary. As a consequence, we can decompose $P(Z|\hat{y})$ into two terms: one which depends on $y_{k}$ and one which does not. For this, we pick a scalar *μ* and rewrite $P(Z|\hat{y})$ as

(19) $$P(Z|\hat{y})=\alpha (y^{\setminus k})+(y_{k}-\mu )\beta (y^{\setminus k}),$$

where $y^{\setminus k}=(y_{1},\ldots ,y_{k-1},y_{k+1},\ldots ,y_{K})$ and

$$\begin{array}{rcl} \alpha (y^{\setminus k}) & = & \mu P(Z|\hat{y}\cup \{t_{k}\})+(1-\mu )P(Z|\hat{y}\setminus \{t_{k}\})\\ \beta (y^{\setminus k}) & = & P(Z|\hat{y}\cup \{t_{k}\})-P(Z|\hat{y}\setminus \{t_{k}\}). \end{array}$$

Plugging Equation 19 into Equation 18 yields ${\mathrm{grad}}_{k}$ as sum of two terms

(20) $$\begin{array}{rcl} {\mathrm{grad}}_{k} & = & A_{k}+B_{k}\;\text{where}\\ A_{k} & = & \int dZ\;\sum_{y}P_{w}(y)\alpha (y^{\setminus k})R(Z)\frac{\partial }{\partial w}\log P_{w}(y_{k})\\ B_{k} & = & \int dZ\;\sum_{y}P_{w}(y)R(Z)(y_{k}-\mu )\beta (y^{\setminus k})\frac{\partial }{\partial w}\log P_{w}(y_{k}). \end{array}$$

Rearranging terms in $A_{k}$, we get

$$A_{k}=\int dZ\;\sum_{y^{\setminus k}}P_{w}(y^{\setminus k})R(Z)\alpha (y^{\setminus k})\sum_{y_{k}}P_{w}(y_{k})\frac{\partial }{\partial w}\log P_{w}(y_{k}).$$

Now, $\sum_{y_{k}}P_{w}(y_{k})\frac{\partial }{\partial w}\log P_{w}(y_{k})=\sum_{y_{k}}\frac{\partial }{\partial w}P_{w}(y_{k})=\frac{\partial }{\partial w}1=0$, hence

(21) $$A_{k}=0\;\text{and}\;{\mathrm{grad}}_{k}=B_{k}.$$

The two equations above encapsulate our main idea for improving on ZR. In showing that $A_{k}=0$ we summed over the two outcomes $y_{k}\in \{0,1\}$, thus identifying a noise contribution in the ZR estimator $R(Z)\frac{\partial }{\partial w}\log P_{w}(y_{k})$ for ${\mathrm{grad}}_{k}$ which vanishes through the averaging by the sampling procedure. Note that the remaining contribution $B_{k}$ has as factor $\beta (y^{\setminus k})$, a term which explicitly reflects how a NMDA-event at time $t_{k}$ contributes to the generation of somatic action potentials. In going from Equation 20 to Equation 21, we assumed that the parameter *μ* was constant. However, a quick perusal of the above derivation shows that this is not really necessary. For justifying Equation 21, one just needs that *μ* does not depend on $y_{k}$, so that $\alpha (y^{\setminus k})$ is indeed independent of $y_{k}$. In the sequel, it shall turn to be useful to introduce a value of *μ* which depends on somatic quantities.

A drawback of Equations 20 and 21 is that they do not immediately lend themselves to Monte-Carlo estimation by sampling the process generating neuronal events. The reason being the missing term $P(Z|\hat{y})$ in the formula for $B_{k}$. To reintroduce the term, we set

(22) $${\tilde{\beta }}_{y}(t_{k})=\beta (y^{\setminus k})/P(Z|y)$$

and in view of Equations 20 and 21 have

$${\mathrm{grad}}_{k}=\int dZ\;\sum_{y}P_{w}(y)P(Z|y)R(Z)(y_{k}-\mu ){\tilde{\beta }}_{y}(t_{k})\frac{\partial }{\partial w}\log P_{w}(y_{k}).$$

Hence, $R(Z)(y_{k}-\mu ){\tilde{\beta }}_{y}(t_{k})\frac{\partial }{\partial w}\log P_{w}(y_{k})$ is an unbiased estimator of ${\mathrm{grad}}_{k}$ and, since ${\mathrm{grad}}_{k}$ gives the contribution to $\frac{\partial }{\partial w}r_{\delta }$ from the *k*th time step,

(23) $$g_{\delta }^{\mathrm{CR}}=R(Z)\sum_{k=1}^{K}(y_{k}-\mu ){\tilde{\beta }}_{y}(t_{k})\frac{\partial }{\partial w}\log P_{w}(y_{k})$$

is an unbiased estimator of $\frac{\partial }{\partial w}r_{\delta }$. Note that, while unavoidable, the above recasting of the gradient calculation as an estimation procedure does seem risky. Due to the division by P(Z|y) in introducing $\tilde{\beta }$, Equation 22, rare somatic spike trains *Z* can potentially lead to large values of the estimator $g_{\delta }^{\mathrm{CR}}$.

To obtain a CR estimator $g^{\mathrm{CR}}$ for the expected reward $\bar{R}$ in our original problem, we now just need to take *δ* to 0 in Equation 23 and tidy up a little. The detailed calculations are presented in Appendix 1, here we just display the final result:

(24) $$\begin{array}{rcl} g^{\mathrm{CR}}(Y,Z) & = & R(Z)\int_{0}^{T}dt\;((1-\mu )(1-e^{-\gamma_{Y}(t)})Y(t)\\ & & +\mu (e^{\gamma_{Y}(t)}-1)q_{N}e^{\beta_{N}u(t)})\beta_{N}\psi (t),\\ \gamma_{Y}(t) & = & \log\frac{P(Z|Y\cup \{t\})}{P(Z|Y\setminus \{t\})}\\ & = & \int_{t}^{\min(T,t+\Delta )}ds\left(1-\Psi_{Y\setminus \{t\}}(s)\right)\\ & & \times \left(a\beta_{S}Z(s)-q_{S}(e^{a\beta_{S}}-1)e^{\beta_{S}U_{\mathrm{base}}(s;Z)}\right). \end{array}$$

In contrast to the ZR-estimator, $g^{\mathrm{CR}}$ depends on somatic quantities via $\gamma_{Y}(t)$ which assesses the effect of having a NMDA-event at time *t* on the probability of the observed somatic spike train. This requires the integration over the duration Δ of a NMDA-spike.

The CR-rule can be written as the sum of two terms, a time-discrete one depending on the NMDA-events Y, and a time-continuous one depending on the instantaneous NMDA-rate, both weighted by the effect of an NMDA-event on the probability of producing the somatic spike train:

$$\begin{array}{rcl} g^{\mathrm{CR}}(Y,Z) & = & \left(1-\mu )R(Z)\int_{0}^{T}dt\;\frac{P(Z|Y\cup \{t\})-P(Z|Y\setminus \{t\})}{P(Z|Y\cup \{t\})}Y(t)\beta_{N}\psi (t\right)\\ & & +\mu R(Z)\int_{0}^{T}dt\;\frac{P(Z|Y\cup \{t\})-P(Z|Y\setminus \{t\})}{P(Z|Y\setminus \{t\})}q_{N}e^{\beta_{N}u(t)}\beta_{N}\psi (t). \end{array}$$

## 5 Performance of zone and cell reinforcements

**Fig. 2:**
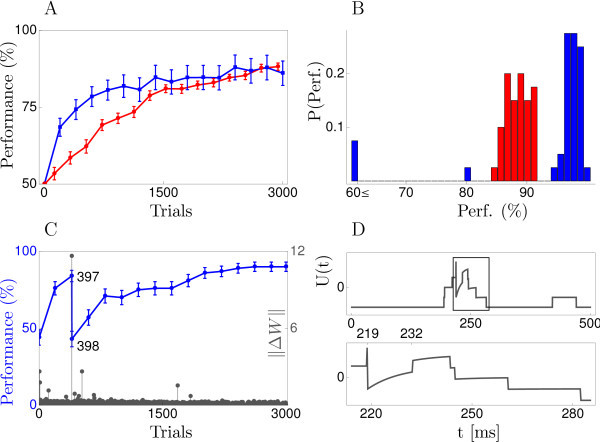
Learning to stay quiescent. **(A)** Learning curves for cell reinforcement (*blue*) and zone reinforcement (*red*) when the neuron should not respond with any somatic firing to one pattern which is repeatedly presented. Values shown are averages over 40 runs with different initial weights and a different input pattern. **(B)** Distributions of the performance after 1500 trials. **(C)** A bad run of the CR-rule where performance drops dramatically after the 397th pattern presentation. The grey points show the Euclidean norm of the change ∥ΔW∥ in the neurons weight matrix *W*, highlighting the excessively large synaptic update after trial 397. **(D)** Time course of the somatic potential during trial 397 (the straight line at t=219 ms marks a somatic spike). As shown more clearly by the blow-up in the bottom row an NMDA-spike occurring at $t^{*}=232\text{ ms}$ yields a value of *U* which stays strongly positive for some 10 ms. (*U* drops thereafter because a NMDA-spike in a different zone ends.) Improbably, however, the sustained elevated value of *U* after $t^{*}$ does not lead to a somatic spike. Hence, the likelihood of the observed somatic response *Z* given the activity $Y^{\nu }$ in the zone *ν* where the NMDA-spike at time $t^{*}$ occurred is quite small, $P(Z_{\left[t^{*},t^{*}+\Delta \right]}|Y^{\nu })=P(Z_{\left[t^{*},t^{*}+\Delta \right]}|Y^{\nu }\cup \{t^{*}\})\approx 0.017$. Indeed, the actual somatic response would have been much more likely without the NMDA-spike, $P(Z_{\left[t_{s},t_{s}+\Delta \right]}|Y^{\nu }\setminus \{t^{*}\})\approx 0.72$. The discrepancy between the two probabilities yields a large value of $\exp(-\gamma_{Y^{\nu }}(t^{*}))$ in Equation 24, leading to the strong weight change. Error bars in the figure show 1 SEM.

To compare the two plasticity rules, we first consider a rudimentary learning scenario where producing a somatic spike during a trial of duration T=500 ms is deemed an incorrect response, resulting in reward R(Z,X)=−1. The correct response is not to spike (Z=∅) and this results in a reward of 0. With these reward signals, synaptic updates become less frequent as performance improves. This compensates somewhat for having a constant learning rate instead of the decreasing schedule which would ensure proper convergence of the stochastic gradient procedure. We use a=0.5 for the NMDA-spike strength in Equation 5, so that just 2-3 concurrent NMDA-spikes are likely to generate a somatic action potential. The input pattern **X** is held fixed and initial weight values are chosen so that correct and incorrect responses are equally likely before learning. Simulation details are given in Appendix 2. Given our choice of *a* and the initial weights, dendritic activity is already fairly low before learning and decreasing it to a very low level is all that is required for good performance in this simple task (Figure 2). 

Simulations for ZR and CR (with a constant value of $\mu =\frac{1}{2}$) are shown in panel 6A. Given the sophistication of the rule, the performance of CR is disappointing, yielding on average only a modest improvement over ZR. The histogram in panel 6B shows that in most cases CR does in fact learn substantially faster than ZR but, in contrast to ZR, CR spectacularly fails on some runs. Performance in a bad run of the CR-rule is shown in panel 6C, revealing that performance can deteriorate in a single trial. In this trial, a very unlikely somatic response was observed (panel 6D), resulting in a large value of $\gamma_{Y}$, thus leading to an excessively large change in synaptic strength.

The finding that large fluctuations in the CR-estimator can arise from rare somatic events, confirms the suspicion in Section 4.2 that recasting Equation 20 as a sampling procedure can lead to problems. Luckily, this can be addressed using the additional degree of freedom provided by the parameter *μ* in the CR-rule. To dampen the effect of the fluctuations in $\gamma_{Y}$, we set *μ* to the time-dependent value

(25) $$\mu =\frac{1}{1+e^{\gamma_{Y}(t)}}=\frac{P(Z|Y\setminus \{t\})}{P(Z|Y\cup \{t\})+P(Z|Y\setminus \{t\})}.$$

Note that *μ* is independent of whether or not t∈Y. Hence, in view of our remark following Equation 21, this is in fact a valid choice for *μ*. The specific form of Equation 25 is to some extent motivated by the aesthetic considerations. It simplifies the first line of Equation 24 to

(26) $$g^{\mathrm{bCR}}(Y,Z)=R(Z)\int_{0}^{T}dt\;\tanh\left(\frac{1}{2}\gamma_{Y}(t)\right)\left(Y(t)+q_{N}e^{\beta_{N}u(t)}\right)\beta_{N}\psi (t).$$

**Fig. 3:**
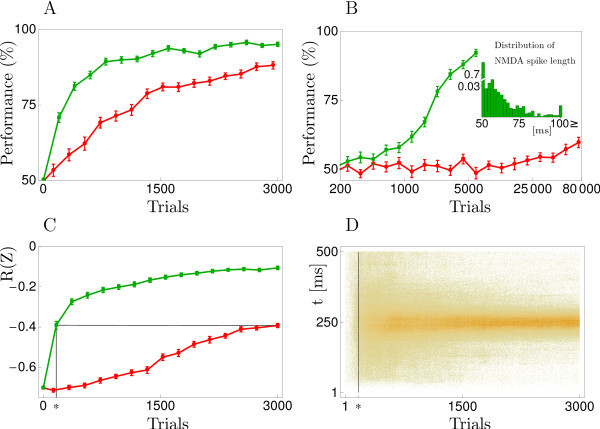
Balanced cell reinforcement (bCR, Equation 26) compared to zone reinforcement. **(A)** Average performance of bCR (*green*) and ZR (*red*) on the same task as in panel 6A. **(B)** Performance when learning stimulus-response associations for four different patterns; bCR (*green*), ZR (*red*), a logarithmic scale is used for the *x*-axis. The inset shows the distribution of NMDA-spike durations after learning the task with bCR. The performance values in the figure are averages over 40 runs, and error bars show 1 SEM. **(C)** Development of the average reward signal R(Z) for bCR (*green*) and ZR (*red*) when the task is to spike at the mid time of the single input pattern ($R(Z)=-2/(nT)\sum_{i}|t_{i}^{\mathrm{sp}}-t^{\mathrm{targ}}|$, where $t_{i}^{\mathrm{sp}}\in Z$, i=1,…,n, is the *i*th of the *n* output spike times, $t^{\mathrm{targ}}=250\text{ ms}$ the target spike time, and T=500 ms the pattern duration; if there was no output spike within [0,T) we added one at *T*, yielding R(Z)=−1). **(D)** Spike raster plot of the output spike times *Z* with R(Z) shown in C using bCR. With ZR, the distribution of spike times after 3000 trials roughly corresponds to the one for bCR after 160 trials (vertical line at ∗), where the two performances coincide (see ∗ and *black lines* in C). The mean and standard deviation of the spike times at the end of the learning process, averaged across the last 300 trials, was 251±45 and 256±121 ms for bCR and ZR, respectively.

We refer to this estimator as balanced cell reinforcement (bCR) (Figure 3). 

From the third line of Equation 24, one sees that the somato-dendritic interaction term in Equation 26 can be written as $\tanh(\frac{1}{2}\gamma_{Y}(t))=\frac{P(Z|Y\cup \{t\})-P(Z|Y\setminus \{t\})}{P(Z|Y\cup \{t\})+P(Z|Y\setminus \{t\})}$. This highlights the terms role as assessing the relevance to the produced somatic spike train of having an NMDA-event at time *t*. In this, it is analogous to the $e^{\pm \gamma_{Y}}$ terms in the CR-rule. But in contrast to these terms, $\tanh(\frac{1}{2}\gamma_{Y})$ is bounded. In ZR, plasticity is driven by the exploration inherent in the stochasticity of NMDA-event generation. Formally, this is reflected by the difference $Y(t)-q_{N}e^{\beta_{N}u(t)}$ entering as a factor in Equation 15, which represents the deviation of the sampled NMDA-events from the expected rate. In bCR, this difference has become a sum. Hence, exploration at the NMDA-event level is only of minor importance for the bCR-rule, where the essential driving force for plasticity is the somatic exploration entering through the factor $\tanh(\frac{1}{2}\gamma_{Y})$.

Due to the modification, bCR consistently and markedly improves on ZR, as demonstrated by panel 5A which compares the learning curves for the same task as in panel 6A. The performance improvement seems to become even larger for more demanding tasks. This is highlighted by panel 5B showing the performance when not just one but four different stimulus-response associations have to be learned. For two of the patterns, the correct somatic response was to emit at least one spike, for the other two patterns the correct response was to stay quiescent. One of the four stimulus-response associations was randomly chosen on each trial and, as before, correct somatic responses lead to a reward signal of R=0 whereas incorrect responses resulted in R=−1. The inset to panel 5B shows the distribution of NMDA-spike durations after learning the four stimulus-response associations with bCR. Over 70% of the NMDA-spikes last for just a little longer than the minimal length of Δ=50 ms. Further nearly all of the spikes are shorter than 100 ms, thus staying well within a physiologically reasonable range.

Panels 5C and 5D show results in a task where reward delivery is contingent on an appropriate temporal modulation of the firing rate. Also, in this second output coding paradigm, the bCR-update is found to be much more efficient in estimating the gradient of the expected reward.

## 6 Discussion

We have derived a class of synaptic plasticity rules for reinforcement learning in a complex neuronal cell model with NMDA-mediated dendritic nonlinearities. The novel feature of the rules is that the plasticity response to the external reward signal is shaped by the interaction of global somatic quantities with variables local to the dendritic zone where the nonlinear response to the synaptic release arises. Simulation results show that such so-called CR rules can strongly enhance learning performance compared to the case where the plasticity response is determined just from quantities local to the dendritic zone.

 In the simulations, we have considered only a very simple task with a single complex cell learning stimulus-response associations. The results, however, show that compared to ZR the bCR rule provides a less noisy procedure for estimating the gradient of the log-likelihood of the somatic response given the neuronal input ($\frac{\partial }{\partial w_{i,\nu }}\log P_{w}(Z|X)$). Estimating this gradient for each neuron is also the key step for reinforcement learning in networks of complex cells [13]. Further, simply memorizing the gradient estimator with an eligibility trace until reward information becomes available, yields a learning procedure for partially observable Markov decision processes, i.e., tasks where the somatic response may have an influence on which stimuli are subsequently encountered and where reward delivery may be contingent on producing a sequence of appropriate somatic responses [22-24]. The quality of the gradient estimator is a crucial factor also in these cases. Hence, it is safe to assume that the observed performance advantage of the bCR rules carries over to learning scenarios which are much more complex than the ones considered here.

In this investigation, we have adopted a normative perspective, asking how the different variables arising in a complex neuronal model should interact in shaping the plasticity response - striving for maximal mathematical transparency and not for maximal biological realism. Ultimately, of course, we have to face the question of how instructive the obtained results are for modeling biological reality. The question has two aspects which we will address in turn: (A) Can the quantities shaping the plasticity response be read-out at the synapse? (B) Is the computational structure of the rules feasible?

 (A) The global quantities in CR are the timing of somatic spikes as well as the value of the somatic potential. The fact that somatic spiking can modulate plasticity is well established by STDP experiments (spike timing-dependent plasticity). In fact such experiments can also provide phenomenological evidence for the modulation of synaptic plasticity by the somatic potential, or at least by a low-pass filtered version thereof. The evidence arises from the fact that the synaptic change for multiple spike interactions is not a linear superposition of the plasticity found when pairing a single pre-synaptic and a somatic spike. Explaining the discrepancy seems to require the introduction of the somatic potential as an additional modulating factor [25].

 In CR-learning, however, we assume that the somatic potential *U* (Equation 5) can differ substantially from a local membrane potential $u_{\nu }$ (Equation 1) and both potentials have to be read-out by a synapse located in the *ν*th dendritic zone. In a purely electrophysiological framework, this is nonsensical. The way out is to note that what a synapse in CR-learning really needs is to differentiate between the total current flow into the neuron and the flow resulting from AMPA-releases in its local dendritic NMDA-zone. While the differential contribution of the two flows is going to be indistinguishable in any local potential reading, the difference could conceivably be established from the detailed ionic composition giving rise to the local potential at the synapse. A second, perhaps more likely, option arises when one considers that NMDA-spiking is widely believed to rely on the pre-binding of Glutamate to NMDA-receptors [7]. Hence, $u_{\nu }$ could simply be the level of such NMDA-receptor bound Glutamate, whereas *U* is relatively reliably inferred from the local potential. Such a reinterpretation does not change the basic structure of our model, although it might require adjusting some of the time constants governing the build up of $u_{\nu }$.

 (B) The plasticity rules considered here integrate over the duration *T* corresponding to the period during which somatic activity determines eventual reward delivery. But synapses are unlikely to know when such a period starts and ends. As in previous works [12,18], this can be addressed by replacing the integral by a low-pass filter with a time constant matched to the value of *T*. The CR-rules, however, when evaluating $\gamma_{Y}(t)$ to assess the effect of an NMDA-spike, require a second integration extending from time *t* into the future up to t+Δ. The acausality of integrating into the future can be taken care of by time shifting the integration variable in the first line of Equation 24, and similarly for Equation 26. But the time shifted rules would require each synapse to buffer an impressive number of quantities. Hence, further approximations seem unavoidable and, in this regard, the bCR-rule (Equation 26) seem particularly promising due to its relatively simple structure. Approximating the hyperbolic tangent in the rule by a linear function yields an update which can be written as a proper double integral. This is an important step in obtaining a rule which can be implemented by a biologically reasonable cascade of low-pass filters.

 The derivation of the CR-rules presented above builds on previous work on reinforcement learning in a population of spiking point neurons [18,24,26]. But in contrast to neuronal firings, NMDA-spikes have a non-negligible extended duration and this makes the plasticity problem in our complex cell model more involved. The previous works introduced a feedback signal about the population decision which has a role similar to the somatic feedback in the present CR-rules. A key difference, however, is that the population feedback had to be temporally coarse grained since possible delivery mechanisms such as changing neurotransmitters levels are slow. In a complex cell model, however, a close to instantaneous somatic feedback can be assumed. As a consequence, the CR-rules can now support reinforcement learning also when the precise timing of somatic action potentials is crucial for reward delivery. Yet, if the soma only integrates NMDA-spikes which extend across 50 ms or more, it appears to be difficult to reach a higher temporal precision in the somatic firing. In real neurons, the temporal precision is likely to result from the interaction of NMDA-spikes with AMPA-releases, with the NMDA-spikes determining periods of heightened excitability during which AMPA-releases can easily trigger a precise somatic action potential. While important in terms of neuronal functionality, incorporating the direct somatic effect of AMPA-releases into the model poses no mathematical challenge, just yielding additional plasticity terms similar to the ones for point neurons [20]. To focus on the main mathematical issues, we have not considered such direct somatic effects here.

## Appendix 1

Here, we detail the steps leading from Equation 22 for $g_{\delta }^{\mathrm{CR}}$ to Equation 24 for $g^{\mathrm{CR}}$.

We first obtain a more explicit form for $g_{\delta }^{\mathrm{CR}}$. In view of Equation 22, ${\tilde{\beta }}_{y}(t_{k})=\frac{P(Z|\hat{y}\cup \{t_{k}\})}{P(Z|\hat{y}/\{t_{k}\})}-1$ if $y_{k}=0$, whereas ${\tilde{\beta }}_{y}(t_{k})=1-\frac{P(Z|\hat{y}/\{t_{k}\})}{P(Z|\hat{y}\cup \{t_{k}\})}$ if there is NMDA-triggering at time $t_{k}$. Hence, setting

$$\gamma_{Y}(t)=\log\frac{P(Z|Y\cup \{t\})}{P(Z|Y\setminus \{t\})}\;\text{we have}\;{\tilde{\beta }}_{y}(t_{k})=(2y_{k}-1)\left(1-e^{\gamma_{\hat{y}}(t_{k})(1-2y_{k})}\right)$$

and hence

$$g_{\delta }^{\mathrm{CR}}(Y,Z)=R(Z)\sum_{k=1}^{K}(y_{k}-\mu )(2y_{k}-1)\left(1-e^{\gamma_{\hat{y}}{\left(t_{k}\right)}^{\left(1-2y_{k}\right)}}\right)\frac{\partial }{\partial w}\log P_{w}(y_{k}).$$

Further, from Equation 16,

$$\begin{array}{rcl} \frac{\partial }{\partial w}\log P_{w}(y_{k}=1) & = & \beta_{N}\psi (t_{k})+O(\delta ),\\ \frac{\partial }{\partial w}\log P_{w}(y_{k}=0) & = & -\delta \beta_{N}q_{N}e^{\beta_{N}u(t_{k})}\psi (t_{k}). \end{array}$$

Hence, taking the limit δ→0, we obtain

$$\begin{array}{rcl} g^{\mathrm{CR}}(Y,Z) & = & R(Z)\int_{0}^{T}dt\;\beta_{N}\psi (t)((1-\mu )(1-e^{-\gamma_{Y}(t)})Y(t)\\ & & -q_{N}e^{\beta_{N}u(t)}\mu (1-e^{\gamma_{Y}(t)})), \end{array}$$

equivalent to the first equation in Equation 24.

We next need an explicit expression for $\gamma_{Y}(t)$. Going back to its definition (Equation 24) and using Equations 7 and 12 yields

$$\begin{array}{rcl} \gamma_{Y}(t) & = & \int_{0}^{T}\left(\log(q_{S}e^{\beta_{S}U(s;Z,Y\cup \{t\})})Z(s)-q_{S}e^{\beta_{S}U(s;Z,Y\cup \{t\})}\right)\;ds\\ & & -\int_{0}^{T}\left(\log(q_{S}e^{\beta_{S}U(s;Z,Y\setminus \{t\})})Z(s)-q_{S}e^{\beta_{S}U(s;Z,Y\setminus \{t\})}\right)\;ds\\ & = & \int_{0}^{T}\beta_{S}\left(U(s;Z,Y\cup \{t\})-U(s;Z,Y\setminus \{t\})\right)Z(s)\;ds\\ & & -\int_{0}^{T}q_{S}\left(e^{\beta_{S}U(s;Z,Y\cup \{t\})}-e^{\beta_{S}U(s;Z,Y\setminus \{t\})}\right)\;ds\\ & = & \int_{0}^{T}\beta_{S}a\left(\Psi_{Y\cup \{t\}}(s)-\Psi_{Y\setminus \{t\}}(s)\right)Z(s)\;ds\\ & & -\int_{0}^{T}q_{S}e^{\beta_{S}U_{\mathrm{base}}(s;Z)}\left(e^{\beta_{S}a\Psi_{Y\cup \{t\}}(s)}-e^{\beta_{S}a\Psi_{Y\setminus \{t\}}(s)}\right)\;ds. \end{array}$$

We next note that times *s* outside of the interval [t,t+Δ] do not contribute to the above integrals since $\Psi_{Y\cup \{t\}}(s)=\Psi_{Y\setminus \{t\}}(s)$ for such *s*. Further, $\Psi_{Y\cup \{t\}}(s)=1$ for s∈[t,t+Δ]. Hence,

$$\begin{array}{rcl} \gamma_{Y}(t) & = & \int_{t}^{\min(T,t+\Delta )}ds\;a\beta_{S}\left(1-\Psi_{Y\setminus \{t\}}(s)\right)Z(s)\\ & & -q_{S}e^{\beta_{S}U_{\mathrm{base}}(s;Z)}\left[e^{a\beta_{S}}-e^{a\beta_{S}\Psi_{Y\setminus \{t\}}(s)}\right]. \end{array}$$

For the term in square brackets we note that, since $\Psi_{Y\setminus \{t\}}(s)$ is zero or one, $e^{a\beta_{S}}-e^{a\beta_{S}\Psi_{Y\setminus \{t\}}(s)}=e^{a\beta_{S}}-(1-\Psi_{Y\setminus \{t\}}(s)+e^{a\beta_{S}}\Psi_{Y\setminus \{t\}}(s))=(e^{a\beta_{S}}-1)(1-\Psi_{Y\setminus \{t\}}(s))$. Hence, finally,

$$\gamma_{Y}(t)=\int_{t}^{\min(T,t+\Delta )}ds\;\left(1-\Psi_{Y\setminus \{t\}}(s)\right)\left(a\beta_{S}Z(s)-q_{S}(e^{a\beta_{S}}-1)e^{\beta_{S}U_{\mathrm{base}}(s;Z)}\right)$$

which gives the last line of (Equation 24).

## Appendix 2

Here, we provide the remaining simulation details.

An input pattern has a duration of T=500 ms and is made up from 150 fixed spike trains chosen independently from a Poisson process with a mean firing rate of 6 Hz (independent realizations are used for each pattern). We think of the input as being generated by an input layer with 150 sites, with each NMDA-zone having a 50% probability of being connected to one of the sites. Hence, on average a NMDA-zone receives 75 input spike trains and 37.5 spike trains are shared between any two NMDA-zones.

A roughly optimized learning rate was used for all tasks and learning rules. Roughly, optimized means that the used learning rate $\eta^{*}$ yields a performance which is better that when using $1.5\eta^{*}$ or $\eta^{*}/1.5$.

In obtaining the learning curves, for each run a moving average of the actual trial by trial performance was computed using an exponential filter with time constant 0.1. Mean learning curves where subsequently obtained by averaging over 40 runs. The exception to this is the single run learning curve in panel 6C. There, subsequently to each learning trial, 100 non-learning trials were used for estimating mean performance.

Initial weights for each run were picked independently from a Gaussian with mean and variance equal to 0.5. Euler’s method with a time step of 0.2 ms was used for numerically integrating the differential equations.

## Competing interests

The authors declare that they have no competing interests.
